# Antiviral Activity of Compound L3 against Dengue and Zika Viruses In Vitro and In Vivo

**DOI:** 10.3390/ijms21114050

**Published:** 2020-06-05

**Authors:** Fu-Kai Chuang, Ching-Len Liao, Ming-Kuan Hu, Yi-Lin Chiu, An-Rong Lee, Shih-Ming Huang, Yu-Lung Chiu, Pei-Ling Tsai, Bo-Cyuan Su, Tsung-Hsien Chang, Chang-Chi Lin, Chih-Chin Shih, Li-Chen Yen

**Affiliations:** 1 Penghu Branch of Tri-Service General Hospital, Penghu 88056, Taiwan; m870957@gmail.com; 2 Department of Microbiology and Immunology, National Defense Medical Center, 161, Sec. 6, Min-Chuan E. Road, Neihu, Taipei 11490, Taiwan; chinglen@gmail.com (C.-L.L.); a1b2c3d37@gmail.com (P.-L.T.); qscez5995@gmail.com (B.-C.S.); changth@mail.ndmctsgh.edu.tw (T.-H.C.); chalin1@ms38.hinet.net (C.-C.L.); 3 National Institute of Infectious Diseases and Vaccinology, National Health Research Institutes, Miaoli 35053, Taiwan; 4 School of Pharmacy, National Defense Medical Center, Taipei 11490, Taiwan; hmk@mail.ndmctsgh.edu.tw (M.-K.H.); 416806@gmail.com (A.-R.L.); 5 Department of Biochemistry, National Defense Medical Center, Taipei 11490, Taiwan; yc566@georgetown.edu (Y.-L.C.); shihming7102@gmail.com (S.-M.H.); 6 School of Public Health, National Defense Medical Center, Taipei 11490, Taiwan; long_ruth0624@mail.ndmctsgh.edu.tw; 7 Institute of Preventive Medicine, National Defense Medical Center, Taipei 11490, Taiwan; 8 Department of Pharmacology, National Defense Medical Center, Taipei 11490, Taiwan; 898010201@mail.ndmctsgh.edu.tw

**Keywords:** flavivirus, dengue virus, Zika virus, anti-viral drugs, tyrosine kinase inhibitors

## Abstract

Dengue virus (DENV) and Zika virus (ZIKV) are mosquito-borne flaviviruses that cause severe illness after infection. Currently, there are no specific or effective treatments against DENV and ZIKV. Previous studies have shown that tyrosine kinase activities and signal transduction are involved in flavivirus replication, suggesting a potential therapeutic strategy for DENV and ZIKV. In this study, we found that compound **L3** can significantly reduce viral protein expression and viral titers in HEK-293, MCF-7, HepG2, and Huh-7 cells and exhibits superior therapeutic efficacy against flaviviral infection compared to other tyrosine kinase inhibitors. In addition, compound **L3** can decrease endogenous HER2 activation and inhibit the phosphorylation of the HER2 downstream signaling molecules Src and ERK1/2, the levels of which have been associated with viral protein expression in MCF-7 cells. Moreover, silencing HER2 diminished DENV-2 and ZIKV expression in MCF-7 cells, which suggests that HER2 activity is involved in flavivirus replication. Furthermore, in DENV-2-infected AG129 mice, treatment with compound **L3** increased the survival rates and reduced the viremia levels. Overall, compound **L3** demonstrates therapeutic efficacy both in vitro and in vivo and could be developed as a promising antiviral drug against emerging flaviviruses or for concurrent DENV and ZIKV outbreaks.

## 1. Introduction

Flaviviruses comprise several medically important viruses, including Japanese encephalitis virus, West Nile virus, dengue virus (DENV), yellow fever virus, and Zika virus (ZIKV). Flaviviruses have a single-stranded positive-sense RNA genome encoding a single polyprotein that undergoes cleavage by host and viral proteases to form three structural proteins—capsid (C), precursor membrane/membrane (prM/M), and envelope (E)—and seven non-structural proteins (NS1, NS2A, NS2B, NS3, NS4A, NS4B, and NS5) [1]. Large outbreaks of DENV and ZIKV have occurred recently, leading to many cases of infection and illness ranging from dengue fever (DF) to severe dengue hemorrhagic fever (DHF) and dengue shock syndrome (DSS) [2,3]. During pregnancy, ZIKV infection causes congenital malformations in the fetus, such as microcephaly, and neurological abnormalities in infected adults [4,5]. However, despite decades of effort, there are still no specific antiviral drugs approved for the treatment of DENV and ZIKV infection. Thus, the development of anti-flaviviral drugs is crucially needed to decrease the severity and fatality of these diseases.

Previous studies have reported that tyrosine kinase activity and signal transduction are involved in flavivirus replication [6,7]. For example, epidermal growth factor receptor (EGFR) belongs to the ErbB family of receptor tyrosine kinases (RTKs). Inhibition of the phosphorylation of ERK, a signal downstream of EGFR, has been shown to decrease DENV replication [8]. In addition, TAM receptors (Tyro3, Axl, and Mer) are also RTKs. DENV activates tyrosine kinase activities through TAM receptors to facilitate its own replication when infecting 293T cells [9]. Moreover, the Src kinase family is a family of non-receptor (cytoplasmic) tyrosine kinases including Src, Fyn, and Yes. Src kinases participate in DENV replication and assembly, and DENV infection has been shown to be decreased in Src kinase-deficient cells [10,11].

Inhibitor studies indicate that tyrosine kinase inhibitors (TKIs) exert anti-flaviviral effects [12]. Combined treatment with erlotinib (a first-generation EGFR TKI) and sunitinib (a platelet-derived growth factor receptor inhibitor) effectively suppressed four dengue serotypes [13]. Additionally, dasatinib (a second-generation EGFR TKI and a highly potent Src kinase inhibitor) has been shown to inhibit DENV particle assembly and decrease its replication [14]. However, a mutation in the transmembrane domain 3 of the NS4B protein of DENV-2 was identified in dasatinib-resistant viral mutants [11], which shows that the development of novel TKI compounds to circumvent this issue is required.

Given that second-generation EGFR TKIs, such as afatinib and dasatinib, demonstrate covalent irreversible binding to the Erb-B receptor, which potently inhibits the signaling of all homodimers and heterodimers formed by the EGFR and human epidermal growth factor receptors (HER)-2, HER3, and HER4 [15,16,17], EGFR TKIs are promising as effective therapies. In this study, we characterize a series of afatinib-derivative TKI compounds, evaluate the antiviral effects of these compounds against DENV and ZIKV infection in vitro and in vivo, demonstrate the superior therapeutic efficacy of compound **L3** (compared to other TKIs), identify that HER2 activities are involved in DENV and ZIKV replication, and describe a potential potent therapeutic drug against emerging flaviviral infections.

## 2. Results

### 2.1. Series of Afatinib-Derivative TKI Compounds Exhibiting Antiviral Activities without Cytotoxic Effects

**Figure 1:**
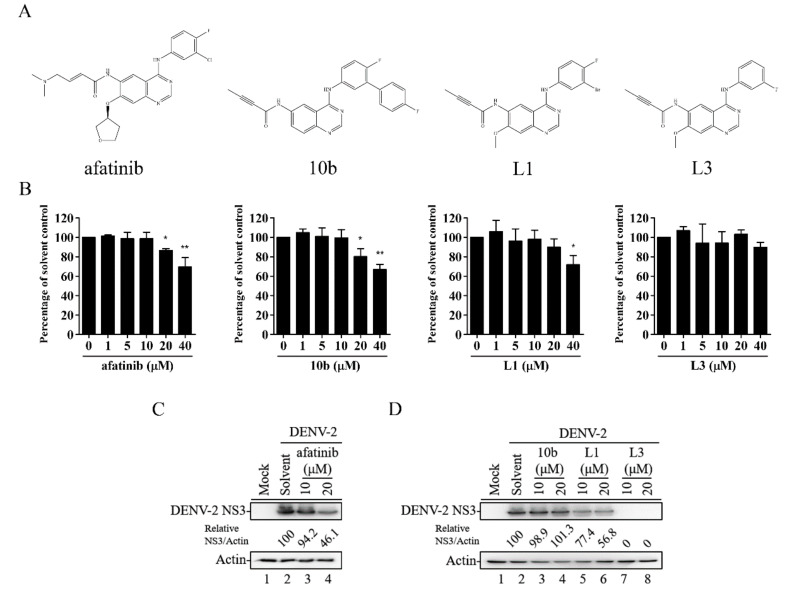
Molecular structures and antiviral abilities of a series of afatinib-derivative tyrosine kinase inhibitor (TKI) compounds. (**A**) Schematic structures of afatinib and compounds **10b**, **L1**, and **L3**. (**B**) HEK-293 cells were treated with a solvent or with different concentrations of afatinib and compounds **10b**, **L1**, and **L3** for 36 h. The WST-1 assay was used to measure cell viability. A percentage was obtained by comparison with the solvent control, set at 100%. (**C**,**D**) HEK-293 cells infected with dengue virus (DENV)-2 (multiplicity of infection (MOI) = 1) and treated with afatinib (**C**) or with compounds **10b**, **L1**, and **L3** (**D**) at 10 and 20 μM. After 36 h, a Western blot analysis of viral protein levels in the cell lysates was performed, and the ratio of the viral NS3 protein level to the level of actin was adjusted to that of the solvent control. Data are the mean ± SD of three independent experiments. * *p* < 0.05, ** *p* < 0.01 according to a two-tailed Student’s *t*-test.

A series of compounds—**10b**, **L1**, and **L3**—were synthesized (Figure 1A) based on the structure of afatinib (which belongs to second-generation irreversible TKIs) [18]. First, we examined the cytotoxic effects of afatinib and compounds **10b**, **L1**, and **L3** in HEK-293 cells using the WST-1 cell proliferation assay. Compared to the other compounds, treatment with compound **L3** did not demonstrate cytotoxicity at concentrations up to 40 μM in HEK-293 cells for 36 h (Figure 1B). Next, we evaluated the antiviral activities of compounds **10b**, **L1**, and **L3** at 10 and 20 μM in DENV-2-infected HEK-293 cells. Treatment with compound **L3** effectively inhibited DENV-2 viral protein expression compared to afatinib (Figure 1C) and compounds **10b** and **L1** (Figure 1D) in HEK-293 cells during DENV-2 infection. Thus, compound **L3** showed potent antiviral ability and was selected for subsequent studies.

### 2.2. Compound ***L3*** Exhibits Antiviral Activity against DENV-1, DENV-2, and ZIKV

**Figure 2:**
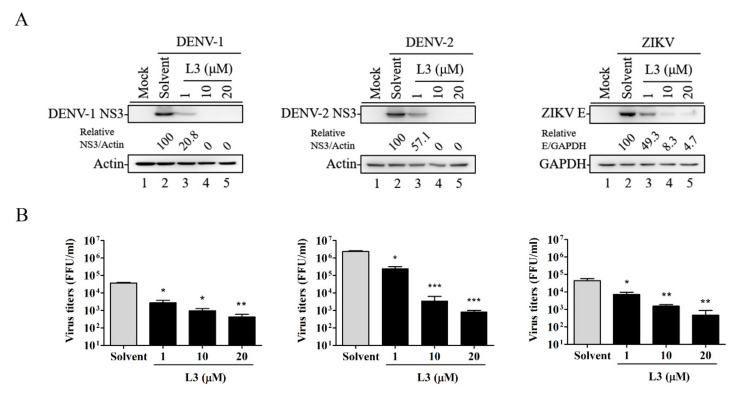
Antiviral activities of compound **L3** against DENV-1, DENV-2, and ZIKV in HEK-293 cells. HEK-293 cells were infected with DENV-1, -2, or ZIKV with or without (solvent) various concentrations of compound **L3** for 36 h. (**A**) Viral protein levels were determined by Western blot analysis. Actin or GAPDH was used as a loading control. Relative ratios of viral NS3 or E protein levels to actin or GAPDH levels were adjusted to those of the solvent control. (**B**) The viral progeny production in the culture supernatants was measured by a focus-forming assay. Data are the mean ± SD of three independent experiments. * *p* < 0.05, ** *p* < 0.01, *** *p* < 0.001 according to a two-tailed Student’s *t*-test.

To determine whether compound **L3** has antiviral activity against DENV and ZIKV, we infected HEK-293 cells with DENV-1, DENV-2, or ZIKV (multiplicity of infection (MOI) = 1) and then treated the cells with different concentrations of compound **L3** for 36 h. As shown in Figure 2A,B, compound **L3** significantly inhibited viral protein expression and viral titers in a dose-dependent manner. In addition, we determined the selectivity index (SI) of compound **L3** for DENV-1, DENV-2, and ZIKV in HEK-293 cells (Table 1). The 50% inhibitory concentration (IC_50_, calculated as the concentration of the drug at which the virus yield was inhibited by 50%) of compound **L3** against DENV-1, DENV-2, and ZIKV in HEK-293 cells at 36 h ranged from 1.8 to 2.3 μM by calculating viral titer levels (Table 1), whereas the 50% cytotoxic concentration (CC_50_, calculated as the concentration that resulted in 50% cellular cytotoxic effect) of compound **L3** in uninfected HEK-293 cells was 61.4 μM at 36 h (Table 1). Thus, the SIs (SI = CC_50_/IC_50_) were 30.7, 26.7, and 34.1 for DENV-1, DENV-2, and ZIKV, respectively (Table 1), suggesting that compound **L3** has broad antiviral ability against flavivirus members.

Furthermore, as liver cells are important target cells during DENV infection [19,20,21], we used the human liver cell lines HepG2 and Huh-7 to further determine the antiviral effects of compound **L3**. Compound **L3** at the indicated doses inhibited viral protein expression and reduced viral progeny production (Figure S1). Taken collectively, these results demonstrate that compound **L3** could be a potential therapeutic drug against flaviviral infection.

### 2.3. Compound ***L3*** Shows Therapeutic Efficacy against DENV-2 and ZIKV Compared to Other TKI Inhibitors

**Figure 3:**
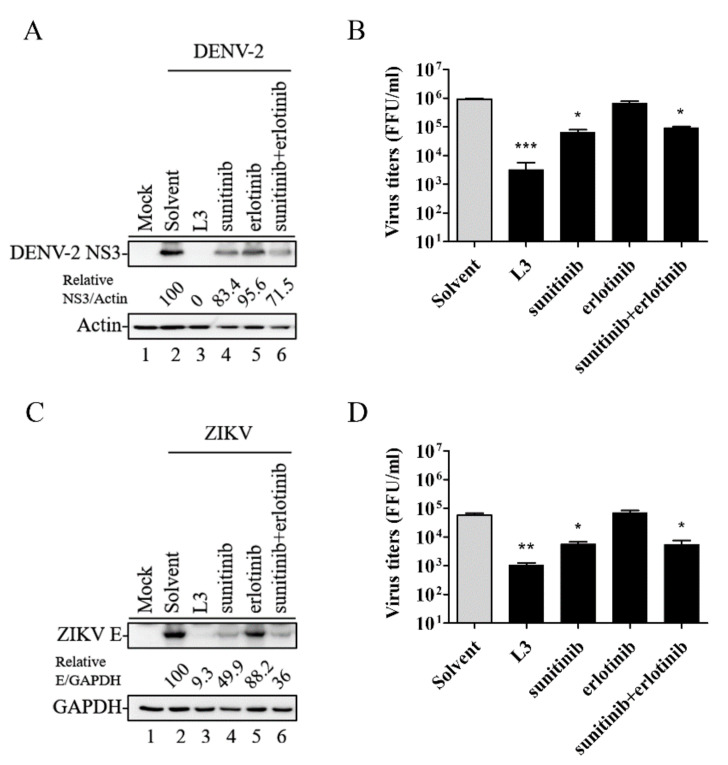
Compound **L3** significantly inhibited DENV-2 or ZIKV compared to other tyrosine kinase inhibitors. HEK-293 cells were infected with DENV-2 (**A**,**B**) or ZIKV (**C**,**D**) and treated with 10 μM of either compound **L3** or the indicated drugs for 36 h. Viral protein expression (**A**,**C**) and virus titers (**B**,**D**) were analyzed and adjusted to those of the solvent control. Data are the mean ± SD of three independent experiments. * *p* < 0.05, ** *p* < 0.01, *** *p* < 0.001 according to a two-tailed Student’s *t*-test.

It has been reported that a combined treatment with erlotinib (a first-generation TKI) and sunitinib can effectively inhibit DENV-2 [13,22]. Thus, we compared the therapeutic efficacy of compound **L3**, sunitinib, erlotinib, and erlotinib plus sunitinib against flaviviral infection. HEK-293 cells were infected with DENV-2 or ZIKV (MOI = 1) and treated with 10 μM of compound **L3**, sunitinib, erlotinib, or erlotinib plus sunitinib for 36 h. As shown in Figure 3A–D, compound **L3** inhibited viral protein expression and reduced viral titers significantly better than the other tyrosine kinase inhibitors, suggesting that compound **L3** had superior anti-flaviviral activity and may have use as a potential therapeutic drug against flaviviral infections. 

### 2.4. Compound ***L3*** Inhibits DENV and ZIKV Replication through the HER2 Signaling Pathway

**Figure 4:**
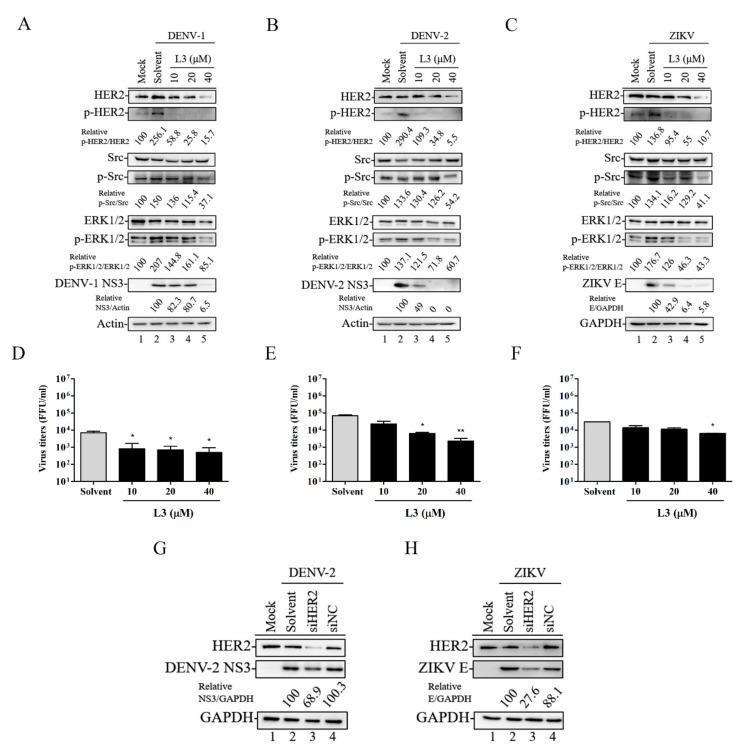
Compound **L3** suppressed DENV and ZIKV replication through the HER2 signaling pathway. (**A**–**C**) MCF-7 cells were infected with DENV-1 for 36 h (**A**), DENV-2 for 30 h (**B**), or ZIKV for 36 h (**C**) (MOI = 1) and treated with 10, 20, or 40 μM of compound **L3** to monitor the phosphorylation of HER2, Src, and ERK1/2 signaling molecules by Western blotting. Relative ratios of *p*-HER2, *p*-Src, and *p*-ERK1/2 levels to HER2, Src, and ERK1/2 levels were adjusted to those of the mock control. Viral protein levels were also determined by Western blot analysis. The relative ratios of viral NS3 or E protein levels to actin or GAPDH levels were adjusted to those of the solvent control. Actin or GAPDH was used as the loading control. (**D**–**F**) Viral titers in culture supernatants were measured by a focus-forming assay. Data are the mean ± SD of three independent experiments. * *p* < 0.05, ** *p* < 0.01 by a two-tailed Student’s *t*-test. (**G**,**H**) MCF-7 cells were transiently transfected with an siRNA-HER2 (siHER2) or an siRNA negative control (siNC). At 24 h after transfection, the cells were infected with DENV-2 (**G**) or ZIKV (**H**) (MOI = 1) for 24 h and lysed for Western blot analysis. GAPDH was used as a loading control. The relative ratios of viral NS3 or E protein levels to GAPDH levels were adjusted to those of the solvent control.

As the TKIs inhibit the activity of HER2 [23], to further explore the antiviral mechanism of compound **L3**, we first used MCF-7 cells that constitutively express endogenous HER2 to investigate whether compound **L3** could reduce flaviviral infection by inhibiting endogenous HER2 activity and HER2 downstream signaling molecules, such as Src and ERK1/2 [24,25]. MCF-7 cells were infected with DENV-1 for 36 h, DENV-2 for 30 h, or ZIKV for 36 h (MOI = 1) and treated with or without 10, 20, or 40 μΜ of compound **L3**. As shown in Figure 4A–C, infection with DENV-1, -2, or ZIKV stimulated HER2, Src, and ERK1/2 phosphorylation (Figure 4A–C, lane 2) compared to mock-infected MCF-7 cells (Figure 4A–C, lane 1). Treatment with compound **L3** reduced the phosphorylated levels of HER2, Src, and ERK1/2 (Figure 4A–C, lanes 3–5) and inhibited viral protein expression (Figure 4A–C) and viral titers (Figure 4D–F) in a dose-dependent manner. Next, we examined whether endogenous HER2 is directly correlated with the modulation of viral replication. We transiently transfected siRNA-HER2 (siHER2) or the siRNA-Negative Control (siNC) into MCF-7 cells for 24 h followed by infection with DENV-2 or ZIKV. As shown in Figure 4G,H, the viral protein expression levels decreased in siHER2 cells compared to siNC cells. Therefore, HER2 activities were critically involved in the machinery of DENV and ZIKV infection, suggesting that HER2 is worthy of further validation as an anti-flaviviral target.

### 2.5. Compound ***L3*** Alleviates DENV-2 Infection in AG129 Mice

**Figure 5:**
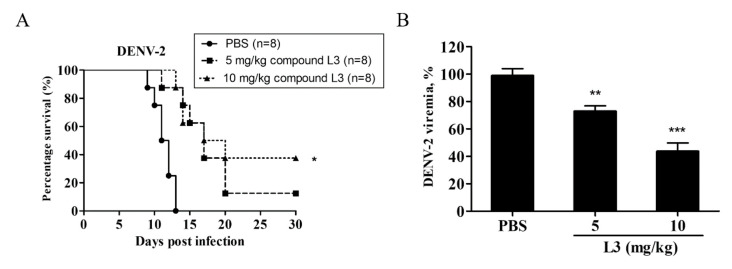
Compound **L3** exhibited protective efficacy in vivo. AG129 mice were infected with 10^7^ focus-forming unit (FFU) of DENV-2 and treated orally with 5 or 10 mg/kg of compound **L3** daily for 7 days. (**A**) Survival rates were determined for 30 days. The number of animals (n) in each group is shown. The data are representative results of three independent experiments. * *p* < 0.05. (**B**) Mouse sera were collected on day 3 after treatment. A focus-forming assay was used to measure DENV-2 viremia. Data are the mean ± SD of three independent experiments. ** *p* < 0.01, *** *p*< 0.001 according to a two-tailed Student’s *t*-test.

To examine the therapeutic potential of compound **L3** against DENV-2 infection in vivo, AG129 mice were infected with 10^7^ FFU DENV-2 and orally treated with compound **L3** at 5 or 10 mg/kg daily for 1 week. The survival rates of the mice treated with 5 and 10 mg/kg of compound **L3** were 12.5% and 37.5%, respectively (Figure 5A). In addition, mouse sera were collected after treatment on day 3 in order to detect viremia. Compound **L3** decreased DENV-2 viremia in a dose-dependent manner (Figure 5B). Overall, compound **L3** improved survival and decreased the viremia levels in DENV-2-infected AG129 mice.

## 3. Discussion

Mosquito-borne flaviviruses, such as DENV and ZIKV, cause hundreds of millions of infections annually and have thus become an increasing public health concern [26,27]. However, there are still no approved antiviral drugs available for the prevention or treatment of DENV and/or ZIKV infection. As several studies have shown that DENV and ZIKV concurrently outbreak in endemic areas [28,29], the development of antiviral agents that act on the host factors are more likely to have pan-antiviral activity, acting not only against DENV but also simultaneously against related flaviviruses, such as ZIKV.

It is well known that flaviviruses regulate intracellular kinases to facilitate viral replication [6,7]. Thus, in this study, we characterized a series of afatinib-derivative TKI compounds (Figure 1) and found that compound **L3** effectively inhibited DENV-1, DENV-2, and ZIKV infection in HEK-293 (Figure 2), MCF-7 (Figure 4), HepG2, and Huh-7 cells (see Figure S1). In addition, compound **L3** inhibited DENV-2 and ZIKV infection in HEK-293 cells, with IC50 values of 2.3 and 1.8 μM (Table 1), whereas the IC50 values of erlotinib against DENV-2 and ZIKV were 2.5 and 6.28 μM in Huh-7 cells, respectively, and the IC50 value of dasatinib against DENV-2 was 4.7 μM in Huh-7 cells [11,13,30]. Although the IC50 values against DENV-2 and ZIKV were calculated in different cell lines (HEK-293 versus Huh-7), compound **L3** exhibited comparable potency with other TKIs, providing a promising anti-flaviviral candidate for further evaluation.

We also compared the therapeutic efficacy of compound **L3** with that of other TKIs (either alone or in combination). Notably, at 10 μM, there were significant reductions in viral proteins and virus titers in DENV-2- and ZIKV-infected HEK-293cells treated with compound **L3** compared to those that underwent sunitinib or erlotinib monotreatment or erlotinib plus sunitinib combination treatment (Figure 3). Thus, compound **L3** may be used (either alone or synergistically with other TKIs) to provide superior treatment against flaviviral infections in the future, especially in concurrent DENV and ZIKV epidemic outbreaks.

We next investigated the mechanism(s) by which compound **L3** inhibited flaviviruses and showed that compound **L3** reduced DENV-1 (at 36 h), DENV-2 (at 30 h), and ZIKV (at 36 h) infection in MCF-7 cells by decreasing HER2 activity and further inhibiting the phosphorylation of the HER2 downstream molecules Src and ERK1/2, thereby suppressing viral protein expression and viral titers (Figure 4A–F). Given that DENV-2 has a higher replication rate than DENV-1 [31,32] and that the tyrosine kinase phosphorylation pattern is dynamic, the detected tyrosine kinase expression levels were still similar but at different time points (36 h versus 30 h) upon DENV-1 or DENV-2 infection (Figure 4A,B). In addition, the observed reduction in viral titer levels was moderate compared to that in the viral protein levels (Figure 2B and Figure 4F and Figure S1B). This possibly occurred because cellular targets contribute to flavivirus replication [12], suggesting that compound **L3** could be further combined with other anti-viral strategies to provide additive or possibly synergistic effects against flavivirus infection. Notably, the viral protein levels of DENV-2 and ZIKV were decreased in HER2-silenced cells, suggesting that HER2 activity is involved in the antiviral mechanism of compound **L3** (Figure 4G,H). Collectively, our findings provide mechanistic insight into the potential role of HER2 signaling in facilitating flavivirus replication and suggest that HER2 may serve as a potential anti-flaviviral therapeutic target. The detailed relationships between HER2 and flaviviruses are worthy of further investigation. 

Previous studies have also shown that liver damage is an important characteristic of dengue fever [19] and that HER2 is also expressed in the human liver cell lines HepG2 and Huh-7 [20,21]. Thus, we further examined the antiviral effects of compound **L3** in HepG2 and Huh-7 cells and found that compound **L3** also significantly inhibited viral protein expression and viral titers during DENV-1 and DENV-2 infection (see Figure S1). Nevertheless, we could not observe the phosphorylation of HER2 upon DENV-2 infection, even though HER2 downstream signaling (i.e., ERK1/2 signaling) was reported to be involved in DENV-induced liver injury [33]. Various host factors and immune responses modulated by DENV-1, DENV-2, or ZIKV need to be characterized in more detail by multiple comparisons of protein samples from flavivirus-infected liver cells collected at different times after treatment with various concentrations of compound **L3**.

Furthermore, a previous study showed that oral treatment with erlotinib at 30 mg/kg once daily did not alter viremia and survival rates, whereas sunitinib at 30 mg/kg or a sunitinib/erlotinib combination treatment once daily for 5 days could reduce the viremia levels on day 2 post-infection and offer partial protection (37%) or greater protection (62%) to DENV-2-infected AG129 mice [13]. In contrast to our study, the oral treatment of compound **L3** at 10 mg/kg daily for 7 days reduced the viremia levels and increased the survival rates (40%) in DENV-2-infected AG129 mice (Figure 5). However, detailed studies on the optimal doses and dosing regimens of compound **L3**, either alone or in combination with other drugs, should be further investigated to improve the compound’s antiviral efficacy in vivo. Overall, our findings reveal that compound **L3** exhibits better therapeutic efficacy in vitro and in vivo compared to other TKIs and can be applied in the treatment of emerging flaviviral infections that lack any identified therapeutic drugs. 

## 4. Materials and Methods

### 4.1. Synthesis of Compounds ***10b***, ***L1***, and ***L3***

*N*-(4-((4′,6-difluoro-[1,1′-biphenyl]-3-yl)amino)quinazolin-6-yl)-2-butynamide was named compound **10b**. The synthesis of compound **10b** was as follows: 2-butynoic acid (0.25 g, 3 mmol) was stirred in THF (10 mL). To this was added *N*-methylmorpholine (0.39 mL, 3.5 mmol), and the mixture was cooled to −10 °C. Isobutylchloroformate (0.39 mL, 3 mmol) was then added, and the mixture was stirred for 0.5 h. *N*^4^-(4′,6-difluoro-[1,1′-biphenyl]-3-yl)quinazoline-4,6-diamine (0.50 g, 1.44 mmol) was dissolved in pyridine (6 mL) and added dropwise to the reaction mixture, which was stirred overnight. The reaction was evaporated to give the crude product, which was purified by column chromatography (EA: 100%) to give compound **10b** (0.19 g, 31.6%): ^1^H-NMR (400 MHz, DMSO-*d*_6_) δ (ppm) 2.05 (3H, s, C-H), 7.31 (3H, m, Ar-H), 7.59 (2H, dd, *J* = 7.4, 6 Hz, Ar-H), 7.74 (2H, m, Ar-H), 7.80 (1H, m, Ar-H), 7.90 (1H, d, *J* = 5.2 Hz, Ar-H), 8.48 (1H, s, Ar-H), 8.72 (1H, s, Ar-H), 9.88 (1H, s, N-H), 10.90 (1H, s, N-H); ^13^C-NMR (100 MHz, DMSO-*d*_6_) δ (ppm) 3.72, 76.12, 85.36, 113.14, 115.67, 115.94, 116.16, 116.27, 116.51, 124.17, 125.04, 127.36, 128.84, 131.20, 131.92, 136.42, 147.18, 151.19, 153.91, 154.41, 156.82, 158.03, 161.09, 163.52; UV λmax (DMSO) nm: 288; m.p.: decomposed at 240–260 °C, IR (KBr) cm^−1^: 3675, 2987, 2900, 1672, 1579, 1546, 1498, 1406, 1393, 1383, 1250, 1241, 1299, 1065, 1027, 891, 828; FDMS: 414.1 [M]^+^; HRFDMS, calculated for C_24_H_16_F_2_N_4_O[M]^+^ 414.1271, found 414.1287. *N*-[4-[(3-Bromo-4-fluorophenyl)amino]-7-methoxyquinazolin-6-yl]-2-butynamide was named compound **L1**. The synthesis of compound **L1** was as follows: 2-butynoic acid (0.25 g, 3 mmol) was stirred in THF (10 mL). To this was added *N*-methylmorpholine (0.39 mL, 3.5 mmol), and the mixture was cooled to −10 °C. Isobutylchloroformate (0.39 mL, 3 mmol) was added, and the mixture was stirred for 0.5 h. *N*-(3-bromo-4-fluorophenyl)-7-methoxyquinazoline-4,6-diamine (0.37 g, 1.03 mmol) was dissolved in pyridine (6 mL) and added dropwise to the reaction mixture, which was stirred overnight. The reaction was evaporated to give the crude product, which was purified by column chromatography to give compound **L1** (0.17 g, 34%): ^1^H-NMR (400 MHz, DMSO-*d*_6_) δ2.05 (3H, s, C-H3), 3.96 (3H, s,C-H3), 7.26 (1H, s, Ar-H), 7.35 (1H, t, *J* = 8.4 Hz, Ar-H), 7.83 (1H, s, Ar-H), 8.21 (1H, d, *J* = 3.6 Hz, Ar-H), 8.5 3(1H, s, Ar-H), 8.64 (1H, s, Ar-H), 9.76 (1H, s, N-H), 10.06 (1H, s, N-H); ^13^C-NMR (100 MHz, DMSO-*d*_6_) δ3.31, 56.22, 75.52, 85.06, 106.95, 107.03, 107.24, 108.65, 116.14, 116.37, 123.04, 123.11, 126.28, 136.90, 149.66, 151.30, 153.08, 154.31, 155.48, 156.82.; UV λmax (DMSO) nm: 289: m.p.: decomposed at 240–260 °C; IR (KBr) cm^−1^: 2242, 1672, 1580, 1550, 1510 1431; LRFDMS: [M]^+^ 428.10; HRFDMS, calculated for C_19_H_14_BrFN_4_O_2_[M]^+^ 428.0278, found 428.0289. *N*-(4-((3-fluorophenyl)amino)-7-methoxyquinazolin-6-yl)but-2-ynamide was named compound **L3**. The synthesis of compound **L3** was as follows: 2-butynoic acid (0.25 g, 3 mmol) and *N*-methylmorpholine (0.39 mL, 3.5 mmol) were stirred in THF (10 mL). After the solution was cooled to −10 °C, isobutylchloroformate (0.39 mL, 3 mmol) was added, and the mixture was stirred for 0.5 h. *N*-(3-fluorophenyl)-7-methoxyquinazoline-4,6-diamine (0.28 g, 1 mmol) was dissolved in pyridine (6 mL) and added dropwise to the reaction mixture, which was stirred overnight. The reaction mixture was evaporated to give a crude residue and was purified by column chromatography to give compound **L3** (0.13 g, 31%): ^1^H-NMR (400 MHz, DMSO-*d*_6_) δ2.05 (3H, s, C-H_3_), 3.97 (3H, s, C-H_3_), 6.88 (1H, t, *J* = 8, 6.8 Hz, Ar-H), 7.27 (1H, s, Ar-H), 7.35 (1H, dd, *J* = 8, 7.6 Hz, Ar-H), 7.62 (1H, d, *J* = 8.4 Hz, Ar-H), 7.86 (1H, d, *J* = 11.6 Hz, Ar-H), 8.56 (1H, s, Ar-H), 8.67 (1H, s, Ar-H), 9.78 (1H, s, N-H), 10.06 (1H, s, N-H); ^13^C-NMR (100 MHz, DMSO-*d*_6_) δ3.29, 56.21, 75.25, 85.01, 106.97, 108.42, 108.84, 109.66, 117.46, 117.48, 118.46, 126.05, 141.26, 149.80, 151.33, 154.33, 156.22, 160.77, 163.71; UV λmax (DMSO) nm: 289; m.p.: decomposed at 240–260 °C; IR (KBr) cm^−1^: 2359, 2242, 1672, 1580, 1550, 1510 1431; HRFDMS, calculated for C_19_H_15_FN_4_O_2_[M]^+^ 350.3527, found 350.3521. The purities of compounds **10b**, **L1**, and **L3** (95%) were assessed using an HPLC–UV/MS platform. Afatinib was obtained from Sigma-Aldrich, St. Louis, MO, USA. All drugs were dissolved in DMSO and stored at −20 °C until use.

### 4.2. Cell Lines and Viruses

The human embryonic kidney cell line HEK-293 (ATCC CRL-1573) and the human breast cancer cell line MCF-7 (ATCC HTB-22) were cultured in Dulbecco’s modified Eagle’s medium containing 10% fetal bovine serum (FBS; Thermo Scientific, Waltham, MA, USA). The baby hamster kidney cell line BHK-21 (ATCC CCL-10) and the mosquito cell line C6/36 (ATCC CRL-1660) were grown in RPMI1640 medium containing 5% FBS. The African green monkey kidney cell line Vero (ATCC CCL-81) were maintained in MEM containing 10% FBS. Two serotypes of DENV (DENV-1, Hawaii strain and DENV-2, 16681 strain) were amplified in C6/36 cells. Viral titers were measured using a focus-forming assay in BHK-21 cells, as described previously [34]. The ZIKV PRVABC59 strain was propagated in C6/36 cells. Viral titers were determined using a focus-forming assay in Vero cells, as described previously [35,36].

### 4.3. Drug Cytotoxicity Assay

HEK-293 cells were treated with afatinib and compounds **10b**, **L1**, and **L3** at the indicated doses for 36 h and analyzed using the cell proliferation reagent WST-1 [2-(4-iodophenyl)-3-(4-nitrophenyl)-5-(2,4-disulfophenyl)-2H-tetrazoliumwater-soluble tetrazolium salt] (Roche Molecular Biochemicals, Basel, Switzerland). Samples’ absorbance of WST-1 was determined using an ELISA reader (Molecular Devices LLC, San Jose, CA, USA) at 450 nm according to the manufacturer’s instructions.

### 4.4. Western Blotting

To investigate the antiviral effects of afatinib, erlotinib, sunitinib, and compounds **10b**, **L1**, and **L3**, HEK-293 or MCF-7 cells were infected with DENV-1, -2, or ZIKV with or without (i.e., only solvent) the compounds. At the indicated time points after infection, the cell lysates were analyzed by Western blot with the antibodies anti-DENV NS3 (GeneTex, Irvine, CA, USA), anti-ZIKV E (GeneTex), anti-HER2 (Cell Signaling Technology, Danvers, MA, USA), anti-HER2 phospho Y877 (Cell signaling), anti-Src (GeneTex), anti-Src phospho Tyr418 (GeneTex), anti-ERK1/2 (Cell Signaling), anti-ERK1/2 phospho Thr/Tyr204 (Cell Signaling), anti-GAPDH (Millipore Corporation. Billerica, MA, USA), and anti-actin (Millipore). Then, the membranes were probed with horseradish peroxidase-conjugated goat anti-mouse IgG secondary antibodies (Jackson ImmunoResearch, Suffolk, UK). The signals were developed by enhanced chemiluminescence (Millipore) and photographed using a Luminescent Image Analyzer (LAS-3000; Fujifilm Corporation, Tokyo, Japan).

### 4.5. HER2 Silencing

The specific siRNA targeting HER2 and the siRNA-Negative control were purchased from Thermo. The sense sequences were as follows: siRNA-HER2 (4390824, Thermo), 5′-GUUGGAUGAUUGACUCUGATT-3′ siRNA-Negative Control (4390843, Thermo). MCF-7 cells were seeded into a six-well plate; then, 20 pmol siRNA-HER2 or the siRNA-Negative control were transfected using the Lipofectamine 3000^®^ transfection reagent (Thermo), according to the manufacturer’s instructions.

### 4.6. Mouse Model

All animal experiments were carried out according to the guidelines outlined by the Council of Agriculture, Executive Yuan, Republic of China, in adherence with the Declaration of Helsinki and U.S. National Institute of Health Guidelines for the treatment of laboratory animals. The animal protocol was approved by the Institutional Animal Care and Use Committee (IACUC) of the National Defense Medical Center (permit no. IACUC-18-158). To examine the therapeutic efficacy of compound **L3** against DENV-2 in vivo, AG129 mice were divided into three groups for treatment (n = 8 per group): (1) intraperitoneal (i.p.) injection with 10^8^ focus-forming units (FFU) of DENV-2 and PBS (vehicle control); (2) i.p. injection with 10^8^ FFU of DENV-2 followed by oral administration of 5 mg/kg of compound **L3**; and (3) i.p. injection with 10^8^ FFU of DENV-2 followed by oral administration of 10 mg/kg of compound **L3**. AG129 mice received the same dose daily for 7 days. The survival of the mice was monitored daily for 30 days. For viremia detection, serum samples were collected on day 3 post-infection, and viral titers were measured using a focus-forming assay.

### 4.7. Statistical Analysis

The GraphPad Prism 5.0 software (GraphPad Software, San Diego, CA, USA) was used for data analysis. The data were analyzed using an unpaired *t*-test and presented as the mean ± SD. Survival curves were analyzed by a log-rank test; *p* < 0.05 was considered statistically significant.

## Figures and Tables

**Table 1:** Cytotoxic concentrations and inhibitory concentrations of compound **L3** applied for 36 h to HEK-293 cells.

Virus	CC_50_^a^ (μM)	IC_50_^b^ (μM)	SI ^c^ (CC_50_/IC_50_)
DENV-1	61.4	2.0	30.7
DENV-2	61.4	2.3	26.7
ZIKV	61.4	1.8	34.1

ZIKV, Zika virus.^a^ CC_50_, cytotoxic concentration, is the concentration that induced 50% of cellular cytotoxicity in uninfected HEK-293 cells. ^b^ IC_50_, inhibitory concentration, is the concentration that reduced the virus titer to 50% in HEK-293 cells infected with the indicated viruses (MOI = 1) and treated with various concentrations of compound **L3**. Viral titers were determined at 36 h post-infection by a focus-forming assay. ^c^ SI, selectivity index, = CC_50_/IC_50_.

## Supplementary Materials

Supplementary materials can be found at https://www.mdpi.com/1422-0067/21/11/4050/s1. Figure S1 Antiviral activities of compound L 3 against DENV infection in HepG 2 and Huh 7 cells.
