# Supplementary material for: Antiviral Activity of Compound L3 against Dengue and Zika Viruses In Vitro and In Vivo
Source: Int J Mol Sci. 2020 Jun 5;21(11):4050. doi: 10.3390/ijms21114050 (PMC7312370; doi:10.3390/ijms21114050)
Supplement: Supplementary file 1 [file ijms-21-04050-s001.pdf]

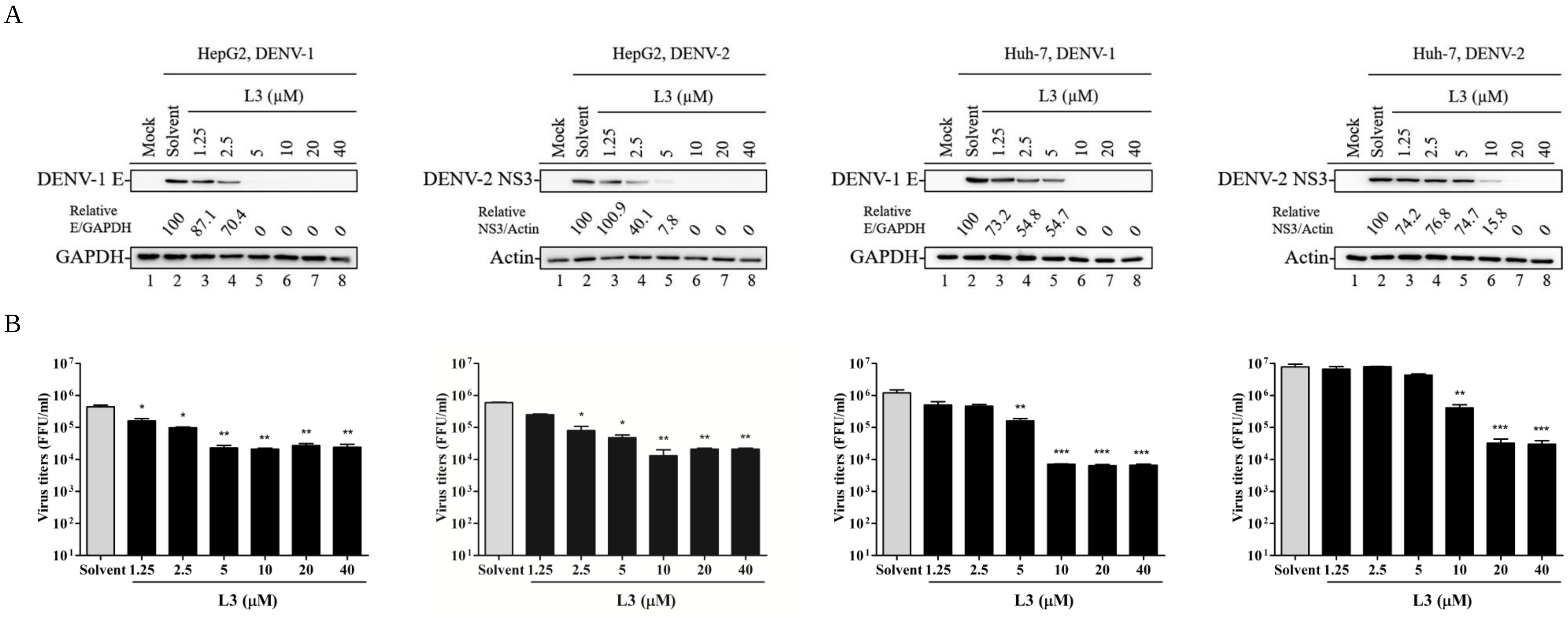

Figure S1. Antiviral activities of compound L3 against DENV infection in HepG2 and Huh-7 cells. (A, B) HepG2 and Huh-7 cells were infected with DENV-1, -2 without (solvent) or with compound L3 for 36 h. (A) Viral protein levels were determined by western blot analysis, and actin or GAPDH was used for a loading control; relative ratios of viral NS3 or E protein levels to actin or GAPDH were adjusted to the solvent control. (B) Viral progeny production in culture supernatants was measured by plaque-forming assay. Data are mean ± SD of 3 independent experiments. * $P < 0.05$, ** $P < 0.01$, ***$P<0.001$ by two-tailed Student t test.
